# Knowledge, Attitudes, and Practices of Spanish Midwives and Midwifery Students toward Oral Healthcare during Pregnancy

**DOI:** 10.3390/ijerph18116089

**Published:** 2021-06-04

**Authors:** Sara Touriño, María del Carmen Suárez-Cotelo, María Jesús Núñez-Iglesias, Eva María Domínguez-Martís, Diego Gabriel Mosteiro-Miguéns, David López-Ares, Silvia Novío

**Affiliations:** 1 Faculty of Nursing, University of Santiago de Compostela, 15782 A Coruña, Spain; sara.tourino@rai.usc.es; 2 Galician Public Healthcare Service, Provincial Hospital of Pontevedra, 36002 Pontevedra, Spain; msuarezcotelo@gmail.com; 3 Department of Psiquiatry, Radiology, Public Health, Nursing and Medicine, University of Santiago de Compostela, 15782 A Coruña, Spain; mjesus.nunez@usc.es; 4 Galician Public Healthcare Service, Healthcare Centre of Concepción Arenal, C/Santiago León de Caracas 12, 15701 A Coruña, Spain; eva.maria.dominguez.martis@sergas.es; 5 Galician Public Healthcare Service, University Hospital Complex of Santiago de Compostela (CHUS), 15706 A Coruña, Spain; diegomoste@gmail.com; 6 Galician Public Healthcare Service, University Hospital Complex of A Coruña (CHUAC), 15006 A Coruña, Spain; dlopares@gmail.com

**Keywords:** antenatal, attitude, knowledge, midwife, oral health, perinatal, practice, pregnancy, students

## Abstract

Pregnancy can affect the mother’s oral health, increasing their susceptibility to oral diseases that have been associated with harmful effects on the newborn. Despite the severity of oral diseases during pregnancy, the demand for dental care during the gestational period is low, which may improve with the participation of midwives in promoting oral health activities. The objectives of this study were: (i) to determine the knowledge, attitudes, and practices of Spanish midwives and midwifery students regarding oral health in pregnant women; and (ii) to identify the barriers faced by these healthcare professionals in addressing oral health promotion during pregnancy. An observational cross-sectional descriptive study was conducted. A total of 128 midwives and/or midwifery students ≥ 18 years old and of both sexes were invited to self-complete a questionnaire between January and April 2020. A total of 85 people participated in the study. Participants had a regular level of knowledge about oral health during pregnancy (overall knowledge score: 6.53), and although they were interested in activities that promote oral healthcare, their oral healthcare practices during pregnancy were limited. As midwives play an important role in promoting health, their training in oral healthcare could help to improve pregnant women’s oral health.

## 1. Introduction

Pregnancy is a normal state that encompasses conception to birth. It involves multiple physiological changes, which can negatively affect oral health [1,2]. It is estimated that around 60% of pregnant women experience oral health problems during pregnancy [3], with a growing prevalence as the pregnancy advances [4].

Hormonal changes and changes in eating habits (for example, the regular consumption of sugary food to fulfill cravings), apart from other factors such morning sickness, increase susceptibility to tooth decay [4,5]. Other oral diseases that have been associated with pregnancy are periodontal disease (manifested mainly as gingivitis, with a prevalence of 60–75% in pregnant women [3] and as periodontitis to a lesser extent), perimolysis, and pyogenic granuloma [5,6]. In this regard, good oral hygiene during pregnancy strongly contributes to the control of such diseases, of which pregnant women should be informed [6].

The oral diseases previously mentioned, apart from being a problem for the mother, can also have harmful effects on the fetus and/or newborn [2,5,6,7]. Periodontal disease during pregnancy is a risk factor for preterm delivery (<37 weeks) and low birthweight (<2500 g) [2,5,6]; similarly, it has been associated with the appearance of pre-eclampsia and miscarriage [5]. After birth, cariogenic bacteria from the mother’s oral biofilm such as *Streptococcus mutans* can be transmitted to the child through saliva, increasing the probability of developing tooth decay in infancy [7].

Despite the severity of oral diseases during pregnancy, and although there are specific programs for oral healthcare during pregnancy [8,9,10], in Spain, the demand for dental procedures during the gestational period is low. Specifically, the percentage of Spanish women who attend dental checkups during their pregnancy does not exceed 15% [9]. Regardless of the different reasons referred to in the literature that may contribute to the low demand for dental care during pregnancy, such as the lack of knowledge of the impact that the mother’s oral health can have on the child [11,12,13] or the false belief that dental treatments are not safe during pregnancy [11,13,14,15], amongst others, all the responsibility for oral health of the pregnant women does not rest with the dentist [16]. To target this problem and given its efficiency [17,18,19,20], oral health during gestation and the postpartum period should be promoted by a multidisciplinary team, with midwives playing an important role, given their close contact with pregnant women during the prenatal visits until after they have given birth [3,21]. However, it is not known if these healthcare professionals have the necessary knowledge to monitor the oral healthcare of pregnant women. Thus, the objectives of this study were (i) to determine the knowledge, attitudes, and practices in a sample of Spanish midwives and midwifery students regarding oral health in pregnant women; and (ii) to identify the barriers experienced by these healthcare professionals in addressing oral health promotion during pregnancy.

## 2. Materials and Methods

### 2.1. Ethical and Legal Considerations

The study was performed with the approval of the Midwives’ Teaching Unit of Galicia. After explaining the procedure and the objective of the investigation, we obtained the participants’ consent and explained that their participation was completely voluntary. Pursuant to the Declaration of Helsinki and the Data Protection Act (Organic Law 3/2018), data confidentiality was guaranteed at all times.

### 2.2. Design

A cross-sectional, descriptive, observational study was conducted.

### 2.3. Setting and Participants

Midwifery students of the Midwives’ Teaching Unit of Galicia, based in the University of Santiago de Compostela (USC), and midwives working in Galicia, one of the autonomous communities in Spain, were invited to participate in the study between January and April of 2020.

The investigation included midwifery students and midwives of either sex, 18 years or older, and who voluntarily agreed to participate. Participants who were not pursuing a nursing degree and/or the specialized training course for midwives in Spain were excluded from the study.

The size of the study population was 128 at the time of the research (*n* = 32 midwifery students and *n* = 96 midwives). Maintaining the expected frequency of all variables at 50%, with a precision of ±4%, and with a midwife-to-midwifery student allocation ratio of 3:1, the desirable sample size using a 95% confidence interval was found to be 85. The sample size was dependent on the number of midwifery students (32 per academic course).

### 2.4. Questionnaire Design and Data Collection

The questionnaire was designed according to the advice of healthcare professionals (dental hygienists, dentists, and midwives), from the literature review, and from previously designed questionnaires [11,22]. Conceptual and semantic equivalence was analyzed for each item. A pilot study was conducted with 15 people who did not participate in the final study in order to evaluate the clarity and ease of understanding of the items, as well as the time required to complete the questionnaire. They reported full comprehension of the questions and ease in completing the questionnaire, so only minimal changes were applied following the pilot study.

The questionnaire consisted of 82 items structured into 5 sections (Supplementary Materials, Table S1). The first section included 12 items about sociodemographic characteristics (age, sex, education, occupation, and years of work experience) and other personal data, such as the number of pregnant women treated per week with oral problems. The second section, consisting of 31 questions, assessed knowledge about oral health during pregnancy. The third section measured the attitude of midwifery students and midwives toward oral healthcare during pregnancy using 20 questions with a 5-point Likert scale for each (1 = strongly disagree to 5 = strongly agree). The fourth section included 8 items with a 5-point Likert scale for each (1 = never to 5 = always) to determine the practices of midwifery students and midwives related to prenatal oral healthcare. The last section, consisting of 11 questions with a 5-point Likert scale for each (1 = strongly disagree to 5 = strongly agree), identified the barriers of their practices in this area.

The results regarding knowledge (second section) were dichotomized as true or false by grouping the answer options as described by Inácio et al. [23]. The variable overall knowledge score (OKS) was then estimated for each participant by calculating the proportion of correct answers for the 31 knowledge-based questions and representing this on a scale from 0 to 10 (0: poor knowledge; 10: good knowledge). Finally, the OKS was categorized, using the Stanones scale, into 3 groups: poor (score < 5), regular (score 5–8), and good (score > 8). The questionnaires were anonymous and self-completed between January and April of 2020. Participants were free to omit any questions they did not want to answer. No incentive was offered for completing the questionnaire. The questionnaires were distributed to midwives and/or midwifery students via: (i) the virtual campus of the University of Santiago de Compostela (USC), (ii) emails, and (iii) word-of-mouth communication.

### 2.5. Statistial Analysis

The results are presented as number and percentage, mean and standard deviation, or median and interquartile range. Numerical (Kolmogorov–Smirnov test, skewness, kurtosis, and the relationships among the mean, median, and mode) and visual (Q–Q plot) methods were used to test the normality of the data.

Bivariate analysis was performed using ANOVA and Student’s *t*-tests for continuous variables and chi-square tests for categorical variables. Significance between multiple experimental groups was determined using Tukey’s post hoc analysis. A *p*-value less than 0.05 was considered significant throughout the study. GNU PSPP 0.8.4 (Free Software Foundation Inc., Boston, MA, USA) and Epidat version 4.2 (Xunta de Galicia, Santiago de Compostela, Spain) were used for the statistical processing of the data.

## 3. Results

### 3.1. Description of Sample

A total of 32 midwifery students of the Midwives’ Teaching Unit of Galicia (16 in their first year and 16 in their second year of study) and 96 midwives working in Galicia were invited to participate in the study, with a response rate of 66.41% (75% midwifery students and 63.54% midwives).

Table 1 shows the sociodemographic characteristics and other personal data about the oral healthcare of the participants.

### 3.2. Knowledge about Oral Health during Pregnancy

The answers to questions about participants’ knowledge are shown in Table 2. The OKS was 6.53 (1.48), which reflects a regular level of knowledge about oral health during pregnancy. The worst-known aspects (less than 15% of correct answers) were those related to how: (i) pregnant women can receive more than just emergency dental care (Item 23), (ii) periodontal disease is associated with both stillbirth (Item 20a), and pre-eclampsia (Item 20d).

The OKS was significantly higher for midwives with more working experience (*p* = 0.001). However, no statistically significant differences were found according to age (*p* = 0.349), work sector (*p* = 0.521), employment status (*p* = 0.948), or bachelor nursing degree studied (*p* = 0.165). In relation to Items 13 to 25, the most frequent significant differences (*p* ≤ 0.05) were found according to the employment status and years of work experience. Midwives with more work experience answered the most questions correctly.

### 3.3. Attitudes toward the Promotion of Oral Health during Pregnancy

The answers to questions about the participants’ attitudes toward the promotion of oral health during pregnancy (Items 26–45) are shown in Table 3. In general, participants had a positive attitude toward activities promoting oral health during pregnancy (Items 26–34, 36, 39, and 43–45). Importantly, the most negative attitudes were observed when they were asked about if they though that conducting a dental assessment in pregnant women was outside the routine practices of midwives (Item 35). This answer could have been influenced by the lack of skills to provide advice (Item 37) or to perform dental assessments (Item 38) on pregnant women.

The most frequent significant differences (*p* ≤ 0.05) were found according to the employment status and years of work experience.

### 3.4. Prenatal Oral Healthcare Practices

Prenatal oral healthcare practices were seldom applied among participants, especially those related to practical skills. Less than 3% of participants reported conducting an oral health assessment on pregnant women during prenatal visits (Item 49). No statistically significant differences were found according to the respondents’ age, the sector where they worked, or their years of work experience as a midwife (*p* > 0.05; Table 4). Only minimal significant differences were observed according to the employment status and the bachelor nursing degree studied.

### 3.5. Barriers to Oral Health Promotion during Pregnancy

The main barrier to oral health promotion during pregnancy identified by midwives and midwifery students was that pregnant women do not demand dental care because they believe that receiving any treatment during pregnancy can affect the child (Item 60). On the contrary, the least important barrier was that dental treatment may cause preterm delivery (Item 63). There were almost no significant differences across the groups (age, education, employment status, years of work experience, and the sector where participants worked) (Table 5).

## 4. Discussion

The results of this study revealed that midwives and midwifery students have a regular level of knowledge about oral health during pregnancy, which improved with years of work experience. They are interested in activities that promote oral healthcare during pregnancy; however, their oral healthcare practices during pregnancy are limited, mainly because they fear that dental procedures may have negative side effects on the fetus and/or newborn. To the best of our knowledge, this is the first study to evaluate the views of Spanish midwives and midwifery students toward maternal oral health and their role in providing oral health education, assessment, and referrals as part of antenatal care. Due to the need to determine provider knowledge and attitudes prior to the planning of educational strategies for the prevention of oral diseases during pregnancy, the results of this study will be of considerable interest due to the important role played by midwives in neonatal care.

Pregnant women are susceptible to poor oral health, which may impact the health outcomes of the mother and the baby [1,2,5]. For this reason, during the prenatal period, emergency dental care as well as routine dental care and regular dental checkups are indicated [6]. In general, most of the participants in the current study were aware of the oral manifestations associated with pregnancy (Item 17), and of the fact that pregnancy can exacerbate preexisting dental problems (Item 15), and that it is recommended for the expectant mother to receive preventive dental care (Item 14) and emergency and routine dental care (Items 23 and 25) during pregnancy. This knowledge explains the high percentage of midwives and midwifery students who advised expectant mothers not to delay or postpone their dental appointments until after delivery (Item 50). However, despite more than 80% of the participants indicating that they knew about the relationship between the mother’s and baby’s oral health (Item 13), their knowledge about the association between periodontal disease and adverse pregnancy outcomes was limited (Item 20). In this sense, other studies that were conducted not only with midwives [22,24,25,26,27,28,29,30] but also with other antenatal care providers, such as general practitioners [22,24,31,32], nurses [22,25], and gynecologists/obstetricians [24,27,30,33], have highlighted that they have insufficient knowledge about oral health and its influence on systemic health, despite the incorporation of oral health into health-promoting strategies and practices [34]; moreover, this is highly recommended [35,36]. The high level of specialization required by healthcare professionals together with a population- versus an individual-focused health delivery system have contributed to the separation between oral and general healthcare [37].

Pregnancy provides a unique opportunity to educate and counsel women about health, including oral health, because they are generally receptive to health information, they are motivated to adopt healthy behaviors, and they maintain a closer and longer contact with healthcare professionals than in other periods of their lives [38]. Furthermore, education about oral health during pregnancy not only improves lifelong oral hygiene and the oral health behaviors of mothers, but it also benefits their children’s overall oral health (for example, reducing the risk of developing early dental decay) [39,40]. However, despite this evidence, in Spain, as in other countries [41,42,43], the dental attendance of pregnant women is low, which has been attributed to multiple barriers [13,15,28,44,45], which were identified by participants in the current study (Items 54–64). Even though the approach to these barriers via the implementation of strategies of oral health promotion have contributed to improving the dental attendance of pregnant women [46,47], in Spain, unlike in other developed countries (for example, Australia) [48,49], the issue of maternal oral health during pregnancy continues to be a poorly assessed and treated aspect, which is almost exclusively managed by dentists, with minimum or zero participation from other healthcare professionals [8,10]. This could be due to the expert panels in charge of the development of Spanish clinical practice guidelines and clinical protocols intended for the attention to pregnant women by midwives being composed of gynecologists, obstetricians, pediatricians, general practitioners, psychiatrists, nurses, and midwives [10,50]. Thus, as non-dental professionals do not have a deep knowledge of oral healthcare ([22,24,25,26,27,28,29,31,32,33,41], and the results of the present study), there is an urgent need for the participation of professionals in the field of odontology in their development.

The potential for antenatal care providers, such as general practitioners, midwives, or gynecologists and obstetricians, to implement preventive oral health activities in their practice has been widely recognized in recent years on an international level, as stated in clinical practice guidelines or clinical protocols [51]. Among the three professional profiles to which we previously referred, midwives are the preferred health professionals to deliver strategies on oral health promotion because they have close contact with women, which allows them to break down barriers, dispel myths about the dangers of dental care during pregnancy, and raise awareness about the importance of oral health from before pregnancy up until the postpartum period. Likewise, they have the opportunity to implement early interventions, and to reevaluate if pregnant women have addressed their dental care needs or if they have implemented correct oral hygiene habits in subsequent follow-up visits during pregnancy [44,52,53,54]. Expectant women seek more dental care when the midwife advises them to do so [55], and women approve of midwives providing oral health education and screening, as well as dental referrals during antenatal and postnatal visits [38,45,55]. Nevertheless, in Spain, the participation of midwives in activities that promote oral health is limited, as demonstrated by the results of this study (Items 46–53). Regarding the latter, despite the only practice in oral healthcare that must be carried out by midwives according to the current monitoring protocols during pregnancy is informing women about the importance of good oral hygiene [50], only approximately 25% were interested in the oral hygiene procedures carried out by pregnant women (Item 48). This could be due to the participants believing that they did not have the skills to provide advice to pregnant women about oral healthcare (Item 37), as oral health education is not integrated into the actual undergraduate midwifery curriculum.

In the current study, the participants had a positive attitude toward oral health during pregnancy, as they acknowledged not only the importance of oral healthcare during this period of life (Items 28, 29, and 36) but also the tasks that midwives can undertake to improve pregnant women’s oral health (Items 30, 34, 39, 44, and 45). These same positive attitudes have been found in previous studies conducted not only with midwives [11,30,56] but also with other healthcare professionals, such as general practitioners, dentists, and obstetricians/gynecologists [11,30,57], which may contribute to improving interprofessional collaboration and communication (Item 33) and thus pregnant women’s oral health [16].

Our study included several limitations. The first is related to the sample population. The sample size was small, limiting the generalization of the results; however, the Midwives’ Teaching Unit of Galicia only has 32 students per year, with the demographic characteristics adequately represented in the sample. Although further research at a national level is required to identify whether the study findings are similar in all Spanish Autonomous Communities, it is hoped that this will be the case, as the curricula for a bachelor of midwifery degree are similar across Spain, as they are regulated by the Department of Health. The results of this study may lay the foundations for the design of a training program for midwives, whose pilot phase will begin in Galicia in the academic year of 2021–2022. The health emergency caused by the SARS-CoV-2 pandemic has hindered the distribution of the questionnaire in person, which would have had a negative impact on the participation rate. Third, as participants filled in the questionnaires themselves, there may be some self-report bias. Fourth, the results of the current study may not be extrapolated to midwives who work in the private sector, as they only represented around 10% of the sample. However, this finding is a reflection of the situation in our country, where the majority of the pregnant women attend public health services.

## 5. Conclusions

Midwives need to improve their theoretical and practical training on oral healthcare during pregnancy so that they can, as leaders of multidisciplinary teams, participate in oral health promotion activities during routine follow-up visits with pregnant women.

## Figures and Tables

**Table 1:** Sociodemographic characteristics and other personal data of the study’s participants.

Item	All Participants *n* = 85 *n* (%)
**Item 1. Age**	
<40 years	56 (65.96)
≥40 years	27 (31.91)
DK/NO	2 (2.13)
**Item 2. Sex**	
Male	4 (4.26)
Female	81 (95.74)
**Item 3. Work sector**	
Public setting	78 (91.49)
Private setting	0
Both	7 (8.51)
**Item 4. Workplace**	
Primary care center	22 (25.53)
Hospital	33 (38.3)
Both	30 (36.17)
**Item 5. Employment status**	
Midwifery student	24 (27.66)
Midwife	61 (72.34)
**Item 6. Education**	
Nursing degree after implementation of the Bologna process	31 (36.17)
Nursing degree before implementation of the Bologna process	52 (61.7)
DK/NO	2 (2.13)
**Item 7. Years of experience as a general nurse**	
<1 year	83 (97.87)
2.5 years	2 (2.13)
**Item 8. Years of experience as a midwife**	
<5 years	27 (31.91)
5–9 years	27 (31.91)
≥10 years	31 (36.17)
**Item 9. Have you received formal education/training on oral healthcare during pregnancy?**	
No	74 (87.23)
Yes	11 (12.77)
**Item 10. What is the average number of pregnant women you treat per week with oral problems?**	
None	27 (31.91)
1–5	54 (63.83)
6–10	4 (4.26)
11–15	0
>15	0
**Item 11. Average number of pregnant women with oral problems who are advised to visit a dentist per week**	
None	27 (31.91)
1–5	50 (59.57)
6–10	2 (2.13)
11–15	0
>15	2 (2.13)
DK/NO	4 (4.26)
**Item 12. Do you give any information about oral healthcare** (**for example, brochures**) **to pregnant women during their routine follow-up visits?**	
No	43 (51.06)
Yes	42 (48.94)

Abbreviations: DK/NO. Do not know/no opinion.

**Table 2:** Knowledge about oral health during pregnancy.

Items	Correct Responses *n* (%)
**Item 13.** Maternal oral health can affect the baby’s oral health	71 (82.98)
**Item 14.** Women must receive preventive dental care during pregnancy	85 (100)
**Item 15.** Pregnancy exacerbates preexisting dental problems	78 (91.49)
**Item 16.** Maternal smoking during pregnancy increases the likelihood of children’s caries lesions	33 (38.3)
**Item 17.** Pregnancy has been associated with:	
Item 17a. Periodontal disease: gingivitis and/or periodontitis	80 (93.62)
Item 17b. Pyogenic granuloma	74 (87.23) ^†^
Item 17c. Caries	60 (70.21) ^†,ɸ^
Item 17d. Perimylolysis	60 (70.21) *
Item 17e. Bruxism	22 (25.53)
**Item 18.** During the pregnancy, calcium is drawn out of mother’s teeth for correct development of the baby	49 (57.45)
**Item 19.** Poor maternal oral health can contribute to early childhood decay	49 (57.45)
**Item 20.** Periodontal disease has been associated with:	
Item 20a. Stillbirth	11 (12.77) ^ɸ^
Item 20b. Preterm delivery	51 (59.57) ^†,‡,ɸ^
Item 20c. Miscarriage	36 (42.55) ^†,ɸ^
Item 20d. Pre-eclampsia	9 (10.64) ^ɸ^
Item 20e. Low birthweight	38 (44.68) ^†,ɸ^
**Item 21.** It is unsafe to obtain dental radiographs in pregnant women	20 (23.4)
**Item 22.** These dental procedures are safe during pregnancy:	
Item 22a. Extractions	71 (82.98) ^†,‡,ɸ^
Item 22b. Local anesthetic	80 (93.62)
Item 22c. Root canal	45 (53.19) ^¥^
Item 22d. Scaling and root planning	36 (42.55)
Item 22e. Tartrectomy with ultrasound	58 (68.09)
Item 22f. Oral hygiene with toothbrush and flossing	83 (97.87)
**Item 23.** Pregnant women must receive only emergency dental care	9 (10.64) ^†,ɸ^
**Item 24.** These drugs are safe during pregnancy:	
Item 24a. Paracetamol	85 (100)
Item 24b. Aspirin	45 (53.19)
Item 24c. Non-steroidal anti-inflammatory drugs	18 (21.28) ^†,ɸ^
Item 24d. Amoxicillin	85 (100)
Item 24e. Erythromycin	43 (51.06) ^¥,†,‡,ɸ^
Item 24f. Doxycycline	45 (53.19)
**Item 25.** Elective dental treatment must be delayed until after pregnancy	29 (34.04)
Overall knowledge score (scale from 1 to 10)	6.53 (1.48) ^ɸ^

The correct answers are underlined. The answers were compared according to the age, work sector, employment status, education, and years of work experience of the respondents. Statistical significance (*p* < 0.05) was determined by the chi-square test (Questions 13–25), and ANOVA and Student’s *t*-tests (overall knowledge score). Significant differences were found according to: * age (participants who were aged < 40 years had a better level of knowledge), ^¥^ work sector (participants who worked in the public and private setting had a better level of knowledge than participants who worked in public settings), ^†^ employment status (midwives had a better level of knowledge than midwifery students, except for Item 24c, for which midwifery students had a better level of knowledge), ^‡^ education (participants who studied nursing before the implementation of the Bologna process had a better level of knowledge), and ^ɸ^ years of work experience (midwives with <5 years of experience had a worse level of knowledge, except for Item 24c, for which midwives with <5 years of experience had a better level of knowledge).

**Table 3:** Attitudes toward the promotion of oral health during pregnancy.

Items	All Participants *n* = 85 *n* (%)		
	Disagree	Neutral	Agree
**Item 26.** Oral health education should be integrated into the undergraduate midwifery curriculum	4 (5.26)	10 (11.64)	71 (82.98)
**Item 27.** Clinical practice guidelines for care in pregnancy and the puerperium should include recommendations on maternal oral health promotion	2 (2.13)	4 (4.26)	79 (93.61)
**Item 28.** Awareness of the importance of oral hygiene during pregnancy is essential *	4 (4.26)	7 (8.51)	74 (87.23)
**Item 29.** Maintaining oral health during pregnancy is important	2 (2.13)	0	83 (97.87)
**Item 30.** Midwives need training in oral health during pregnancy because it could be useful for their professional life	2 (2.13)	4 (4.26)	79 (93.62)
**Item 31.** Women must visit a dentist before getting pregnant ^†^	2 (2.13)	9 (10.64)	74 (87.24)
**Item 32.** Pregnant women are more likely to seek dental care if healthcare providers recommend it	2 (2.13)	0	83 (97.87)
**Item 33.** Currently, there is good understanding between midwives and dentists regarding dental care for pregnant women	18 (21.27)	20 (23.4)	47 (55.32)
**Item 34.** Asking pregnant women about their oral health is outside the routine practices of midwives *	49 (57.45)	11 (12.77)	25 (29.78)
**Item 35.** Conducting a dental assessment in pregnant women is outside the routine practices of midwives	11 (12.77)	16 (19.15)	58 (68.09)
**Item 36.** Dental assessments in pregnant women during the prenatal visits are important	6 (7.39)	14 (15.89)	65 (76.59)
**Item 37.** I have the skills to provide advice to pregnant women about oral healthcare ^†,‡^	47 (55.32)	29 (34.04)	9 (10.64)
**Item 38.** I have the skills to perform dental assessments for pregnant women ^‡,₴^	72 (85.11)	9 (10.64)	4 (4.26)
**Item 39.** There is little midwives can do to improve pregnant women’s oral hygiene and oral health	68 (80.85)	13 (14.89)	4 (4.26)
**Item 40.** Pregnant women feel relaxed when midwives conduct oral assessments during antenatal visits	20 (23.41)	38 (44.68)	27 (31.91)
**Item 41.** The link between periodontal disease and preterm birth and/or low birthweight is too tenuous for me to warn pregnant women about ^†,‡^	45 (52.94)	36 (42.55)	4 (4.26)
**Item 42.** The link between dental caries in mothers and in babies is too tenuous for me to warn pregnant women about *^,†,‡^	42 (49.68)	38 (44.43)	5 (6.39)
**Item 43.** I worry that something will go wrong during a pregnancy due to the mother’s oral problems ^†,ɸ,‡^	18 (21.27)	24 (28.66)	43 (49.93)
**Item 44.** I am interested in further information about oral healthcare in pregnant women	4 (4.26)	4 (4.26)	77 (91.49)
**Item 45.** I am interested in further training to provide dental assessments to pregnant women ^ɸ^	7 (8.52)	7 (8.51)	71 (82.98)

The answers were grouped into three categories: agree (strongly agree and agree), neutral, and disagree (strongly disagree and disagree). The answers were compared according to the age, work sector, employment status, education, and years of work experience of the respondents. Statistical significance (*p* < 0.05) was determined by the chi-square test. Significant differences were found according to * age (participants who were aged ≥ 40 years agreed more than participants aged < 40 years), ^†^ employment status (Items 31 and 43: midwives agreed more than midwifery students; Items 41 and 42: midwives disagreed more than midwifery students; Item 37: midwifery students disagreed more than midwives), ^ɸ^ education (Item 43: participants who studied nursing after the implementation of the Bologna process disagreed more than participants who studied nursing before the implementation of the Bologna process; Item 45: participants who studied nursing after implementation of the Bologna process agreed more than participants who studied nursing before its implementation), ^‡^ years of work experience (Items 37, 38, and 43: midwives with <5 years of experience disagreed more than the other participants; Items 41 and 42: midwives with 5–9 years of experience disagreed more than the other participants), and ^₴^ work sector (participants who worked in a public setting disagreed more than participants who worked in public and private settings).

**Table 4:** Prenatal oral healthcare practices.

Items	All Participants *n* = 85 *n* (%)				
	Never	Rarely	Sometimes	Often	Always
**Item 46.** I ask pregnant women about their oral health	14 (17.02)	14 (17.02)	21 (23.4)	14 (17.02)	22 (25.53)
**Item 47.** I discuss the importance of oral health with pregnant women	20 (23.40)	13 (14.89)	26 (31.91)	13 (14.89)	13 (14.89)
**Item 48.** I ask pregnant women about their oral hygiene procedures	16 (19.15)	31 (36.17)	16 (19.15)	13 (14.89)	9 (10.64)
**Item 49.** I conduct oral health assessments on pregnant women during prenatal visits	59 (70.21)	22 (25.53)	2 (2.13)	2 (2.13)	0
**Item 50.** I advise pregnant women to delay dental visits until after pregnancy ^†^	61 (72.34)	9 (10.64)	11 (12.77)	2 (2.13)	2 (2.13)
**Item 51.** I advise pregnant women to go to the dentist before getting pregnant	19 (21.28)	16 (19.15)	14 (17.02)	25 (29.79)	9 (10.64)
**Item 52.** I provide counselling regarding the association of poor periodontal health with negative birth outcomes ^ɸ^	27 (31.91)	20 (23.40)	18 (21.28)	13 (14.89)	5 (6.38)
**Item 53.** I provide counselling regarding caries prevention and transmission from mother to child	33 (38.30)	20 (23.40)	16 (19.15)	7 (8.51)	7 (8.51)

The answers were compared according to the age, work sector, employment status, education, and years of work experience of the respondents. Statistical significance (*p* < 0.05) was determined by the chi-square test. No statistically significant differences were found according to the respondents’ age, sector worked, or years of work experience as a midwife (*p* > 0.05). Only minimal significant differences were observed according to: ^†^ the employment status (midwives advised pregnant women to delay dental visits until after pregnancy more frequently than midwifery students), and ^ɸ^ the bachelor nursing degree studied (participants who studied nursing before the implementation of the Bologna process provided counselling regarding the association of poor periodontal health with negative birth outcomes more frequently than participants who studied nursing after its implementation).

**Table 5:** Barriers to oral health promotion during pregnancy.

Items	All Participants *n* = 85 *n* (%)		
	Disagree	Neutral	Agree
**Item 54.** Midwives cannot provide oral health education to pregnant women because there is not enough time during prenatal appointments	24 (27.66%)	25 (29.79)	36 (42.56)
**Item 55.** Midwives do not know the importance of oral health during pregnancy	25 (29.78%)	22 (25.53)	38 (44.68)
**Item 56.** Spanish clinical practice guidelines for care in pregnancy and the puerperium do not address oral healthcare	16 (19.15)	27 (31.91)	42 (48.94)
**Item 57.** Midwives do not have appropriate knowledge about oral health during pregnancy	21 (24.9)	37 (44.04)	26 (31.06)
**Item 58.** Midwives do not have the skills to provide dental assessments to pregnant women	17 (19.9)	22 (26.28)	46 (53.70)
**Item 59.** Dental treatments for pregnant women are very expensive	31 (36.17)	33 (38.30)	22 (25.53)
**Item 60.** Pregnant women do not demand dental care because they believe that receiving any treatment during pregnancy can affect the child	16 (19.15)	18 (21.28)	51 (59.58)
**Item 61.** Oral healthcare is not a priority for pregnant women	34 (40.43)	18 (21.28)	33 (38.3)
**Item 62.** Dentists are reluctant to treat pregnant women *	30 (35.04)	7 (8.51)	48 (56.31)
**Item 63.** Dental treatments can cause a preterm delivery ^†^	56 (65.83)	26 (30.79)	3 (3.13)
**Item 64.** Recommendations for oral care are not unanimous ^ɸ,‡^	19 (22.15)	38 (44.43)	28 (32.78)

The answers were grouped into three categories: agree (strongly agree and agree), neutral, and disagree (strongly disagree and disagree). The answers were compared according to the age, work sector, employment status, education, and years of work experience of the respondents. Statistical significance (*p* < 0.05) was determined by chi-square test. Significant differences were found according to: * age (participants who were aged ≥ 40 years disagreed more than participants who were aged < 40 years), ^†^ employment status (midwives disagreed more than midwifery students), ^ɸ^ education (participants who studied their nursing degree before the implementation of the Bologna process agreed more than participants who studied their nursing degree after its implementation), and ^‡^ years of work experience (midwives with 5–9 years of experience disagreed more than the other participants). No statistically significant differences were found according to the sector in which they worked.

## Data Availability

The data presented in this study are available on request from the corresponding author (S.N.) upon reasonable request. The data are not publicly available due to restrictions e.g., their containing information could compromise the privacy of participants.

## Supplementary Materials

The following are available online at https://www.mdpi.com/article/10.3390/ijerph18116089/s1. Table S1: Questionnaire “Knowledge, attitudes, and practices of midwives and midwifery students toward oral healthcare during pregnancy”.
