# Supplementary material for: Knowledge, Attitudes, and Practices of Spanish Midwives and Midwifery Students toward Oral Healthcare during Pregnancy
Source: Int J Environ Res Public Health. 2021 Jun 4;18(11):6089. doi: 10.3390/ijerph18116089 (PMC8200230; doi:10.3390/ijerph18116089)
Supplement: Supplementary file 1 [file ijerph-18-06089-s001.zip › ijerph-1225389-supplementary.pdf]

Table S1. Conocimientos, actitudes y prácticas de matronas y residentes en enfermería obstétrico-ginecológica en materia de salud oral en mujeres embarazadas

PRIMERA PARTE: Aspectos sociodemográficos y otros datos de carácter personal.

1. Edad: ____
2. Sexo: Femenino ☐ Masculino ☐
3. Sector en el que trabaja: Público ☐ Privado ☐ Ambos ☐
4. Nivel asistencial en el que trabaja: Atención primaria ☐ Atención especializada ☐ Ambas ☐
5. Situación laboral: Residente ☐ Matrona ☐
6. Formación profesional: Graduado en enfermería ☐ Diplomado en enfermería ☐ Otra ☐ ________________________________
7. Años de experiencia laboral como graduado/diplomado: ___
8. Años de experiencia laboral como matrona: ____
9. ¿Ha recibido formación sobre salud oral en mujeres embarazadas?: SI ☐ NO ☐
10. ¿Cuántas de las mujeres embarazadas que atiende por semana refieren tener problemas de salud oral?: ______ De las mismas,
11. ¿Cuántas deriva al odontólogo? _____
12. ¿Informa o entrega algún material informativo (ej. folletos) sobre "salud oral en el embarazo" en alguna de las consultas de seguimiento a mujeres embarazadas? SI ☐ NO ☐

SEGUNDA PARTE: Conocimientos sobre salud oral en el embarazo. Por favor, indique con una "X" si son verdaderas o falsas las siguientes afirmaciones.

	**Verdadero**	**Falso**	**No sabe/ no contesta**
13. La salud oral de la madre puede influir en la salud del feto	☐	☐	☐
14. Las mujeres deben recibir atención dental preventiva durante el embarazo	☐	☐	☐
15. El embarazo exacerba los problemas dentales existentes en la madre	☐	☐	☐
16. El tabaquismo prenatal materno aumenta el riesgo de caries del futuro bebé	☐	☐	☐
17. El embarazo se asocia con las siguientes manifestaciones orales:			
a. Enfermedad periodontal: gingivitis (inflamación reversible de las encías) y/o periodontitis (destrucción de los tejidos de sostén del diente)	☐	☐	☐
b. Granuloma piógeno o épulis gravídico	☐	☐	☐
c. Caries dental	☐	☐	☐
d. Erosión dentaria	☐	☐	☐
e. Bruxismo (rechinar los dientes)	☐	☐	☐

18. Durante el embarazo, el calcio de los dientes de la madre es liberado para el correcto desarrollo del futuro bebé	☐	☐	☐
19. La mala salud oral materna puede favorecer la aparición de caries dental en el/la niño/a	☐	☐	☐
20. La enfermedad periodontal se asocia con:			
a. Muerte fetal	☐	☐	☐
b. Parto prematuro	☐	☐	☐
c. Aborto espontáneo	☐	☐	☐
d. Preeclampsia	☐	☐	☐
e. Bajo peso al nacer	☐	☐	☐
21. No es seguro obtener radiografías dentales en mujeres embarazadas	☐	☐	☐
22. Los siguientes procedimientos dentales son seguros durante el embarazo:			
a. Extracciones	☐	☐	☐
b. Anestesia local	☐	☐	☐
c. Endodoncia	☐	☐	☐
d. Raspado y alisado radicular	☐	☐	☐
e. Limpieza con ultrasonidos	☐	☐	☐
f. Higiene con cepillo y seda dental	☐	☐	☐
23. Las mujeres embarazadas sólo deben recibir atención dental de emergencia	☐	☐	☐
24. Los siguientes tratamientos farmacológicos son seguros durante el embarazo:			
a. Paracetamol	☐	☐	☐
b. Aspirina	☐	☐	☐
c. AINE (antiinflamatorio no esteroideo)	☐	☐	☐
d. Amoxicilina	☐	☐	☐
e. Eritromicina	☐	☐	☐
f. Doxiciclina	☐	☐	☐
25. El tratamiento dental electivo debe retrasarse hasta después del embarazo	☐	☐	☐

TERCERA PARTE: Actitud relacionada con la promoción de la salud oral durante el embarazo. Por favor, en una escala de 1 a 5, indique su grado de acuerdo o desacuerdo con las siguientes afirmaciones, siendo 1 en total desacuerdo y 5 totalmente de acuerdo.

Desacuerdo ← → **Acuerdo**

	1	2	3	4	5
26. La formación sobre el cuidado de la salud oral debería incluirse en el programa formativo de la especialidad de Enfermería Obstétrico-Ginecológica (matrona)					
27. Las Guías de Práctica Clínica de atención en el embarazo y puerperio deberían incluir aspectos relacionados con la promoción de la salud oral materna					

28. La concienciación sobre la importancia de la higiene prenatal es un aspecto prioritario.	1	2	3	4	5
29. El mantenimiento de una correcta salud oral durante el embarazo es importante	1	2	3	4	5
30. Las matronas necesitan educación sobre salud oral en mujeres embarazadas ya que podría ser útil para su profesión	1	2	3	4	5
31. Las mujeres que quieren tener hijos deben hacer una revisión dental previa al embarazo	1	2	3	4	5
32. Las mujeres embarazadas son más proclives a buscar atención dental si los profesionales sanitarios se lo recomiendan	1	2	3	4	5
33. Actualmente existe una colaboración activa entre matronas y dentistas con respecto al cuidado dental para mujeres embarazadas	1	2	3	4	5
34. Preguntar a las mujeres embarazadas sobre la salud bucal está fuera de la práctica habitual de las matronas	1	2	3	4	5
35. Realizar un examen visual de la boca durante el embarazo está fuera de la práctica habitual de las matronas	1	2	3	4	5
36. Las exploraciones orales de mujeres embarazadas en visitas prenatales son importantes	1	2	3	4	5
37. Estoy segura/o de que tengo los conocimientos necesarios para aconsejar a las mujeres embarazadas sobre salud bucal	1	2	3	4	5
38. Estoy segura/o de que tengo los conocimientos necesarios para realizar exploraciones orales en mujeres embarazadas	1	2	3	4	5
39. Las matronas poco pueden hacer para mejorar la higiene y salud oral de las mujeres embarazadas	1	2	3	4	5
40. Las mujeres embarazadas se sienten cómodas cuando una matrona evalúa la salud bucal durante las consultas prenatales normales	1	2	3	4	5
41. La relación entre enfermedad periodontal y parto prematuro y/o bajo peso al nacer es demasiado débil para advertir a las mujeres embarazadas al respecto	1	2	3	4	5

42. La relación entre caries dental en las madres y en los bebés es demasiado débil para advertir a las mujeres embarazadas al respecto	1	2	3	4	5
43. Me preocupa que algo salga mal en un embarazo como consecuencia de un problema oral de la madre	1	2	3	4	5
44. Estoy interesada/o en obtener más información sobre salud oral en mujeres embarazadas	1	2	3	4	5
45. Estoy interesada/o en adquirir una mayor capacitación para realizar exploraciones orales en mujeres embarazadas	1	2	3	4	5

CUARTA PARTE: Prácticas en promoción de la salud oral en mujeres gestantes. Por favor, señala del 1 al 5 el valor de la escala que más se identifique con tu práctica: 1. Nunca; 2. Casi nunca; 3. A veces; 4. Casi siempre; 5. Siempre.

Nunca ⟵ ⟶ **Siempre**

46. Pregunto a las mujeres embarazadas sobre su salud oral.	1	2	3	4	5
47. Discuto con mujeres embarazadas la importancia de la salud oral.	1	2	3	4	5
48. Pregunto a las mujeres embarazadas sobre sus prácticas de higiene oral.	1	2	3	4	5
49. Realizo una exploración oral de las mujeres embarazadas como parte de la atención prenatal.	1	2	3	4	5
50. Aconsejo a las mujeres embarazadas que retrasen las visitas al dentista hasta después del embarazo.	1	2	3	4	5
51. Aconsejo a las mujeres que visiten al dentista antes de quedarse embarazadas.	1	2	3	4	5
52. Informo a las mujeres embarazadas sobre la asociación entre una mala salud periodontal y resultados de nacimiento negativos.	1	2	3	4	5
53. Informo a las mujeres embarazadas sobre prevención y riesgo de transmisión de caries entre madre e hija/o.	1	2	3	4	5

QUINTA PARTE: Barreras para la promoción del cuidado oral en mujeres embarazadas. Por favor, en una escala de 1 a 5, indique su grado de acuerdo o desacuerdo con las siguientes afirmaciones, siendo 1 en total desacuerdo y 5 totalmente de acuerdo.

	**Desacuerdo** ⟵			⟶ **Acuerdo**	
54. Las consultas prenatales son demasiado breves como para que las matronas hagan educación para la salud oral a mujeres embarazadas	1	2	3	4	5
55. Las matronas desconocen la importancia de la salud bucal durante el embarazo.	1	2	3	4	5
56. Las guías de práctica clínica de atención en el embarazo y puerperio de uso en España no abordan el cuidado de la salud bucal.	1	2	3	4	5
57. Las matronas no tienen la formación necesaria sobre salud oral en el embarazo.	1	2	3	4	5
58. Las matronas no están capacitadas para realizar exploraciones orales en mujeres embarazadas.	1	2	3	4	5
59. Los tratamientos de patologías orales en mujeres embarazadas son muy caros	1	2	3	4	5
60. Las mujeres embarazadas no demandan atención porque creen que hacer algún tratamiento durante el embarazo puede afectar al niño/a	1	2	3	4	5
61. El cuidado de la salud oral no es una prioridad para las mujeres embarazadas	1	2	3	4	5
62. Los odontólogos son reticentes a tratar a las mujeres embarazadas.	1	2	3	4	5
63. Los tratamientos dentales pueden propiciar un parto prematuro.	1	2	3	4	5
64. Las recomendaciones sobre cuidado oral en el embarazo no son unánimes.	1	2	3	4	5
